# Supplementary material for: Synthesis, Structures, and Water Adsorption of Two Coordination Polymers Constructed by M(II) (M = Ni (1) and Zn (2)) with 1,3-Bis(4-Pyridyl)Propane (bpp) and 1,2,4,5-Benzenetetracarboxylate (BT4−) Ligands
Source: Polymers (Basel). 2020 Sep 27;12(10):2222. doi: 10.3390/polym12102222 (PMC7601724; doi:10.3390/polym12102222)
Supplement: Supplementary file 1 [file polymers-12-02222-s001.pdf]

Supplementary Information

Synthesis, Structures, and Water Adsorption of Two Coordination Polymers Constructed by M(II) (M = Ni (1) and Zn (2)) with 1,3-bis(4-pyridyl)propane (bpp) and 1,2,4,5-benzenetetracarboxylate (BT^{4-}) Ligands

Supporting Information

Table S1. Bond lengths (Å) and angles (°) around Co(II) ion in **1**.

Table S2. Related parameters of O–H···O hydrogen bonds in **1**.

Table S3. Bond lengths (Å) and angles (°) around Zn(II) ion in **2**.

Table S4. Related parameters of O–H···O hydrogen bonds in **2**.

Figure S1 (a) Thermogravimetric (TG) measurement of **1**. (b) Powder X-ray diffraction patterns at RT and selected temperatures and its simulation from single-crystal diffraction data of **1**.

Figure S2 (a) Thermogravimetric (TG) measurement of **2**. (b) Powder X-ray diffraction patterns at RT and selected temperatures and its simulation from single-crystal diffraction data of **2**.

Table S1. Bond lengths (Å) and angles (°) around Ni(II) ion in **1**.

Ni(1)–O(5)	2.039(3)	Ni(1)–N(3)	2.063(4)
Ni(1)–N(1)	2.069(4)	Ni(1)–O(11)	2.089(3)
Ni(1)–O(9)	2.095(3)	Ni(1)–O(10)	2.099(4)
Ni(2)–O(1)	2.051(3)	Ni(2)–N(2)	2.060(4)
Ni(2)–N(4)	2.083(4)	Ni(2)–O(13)	2.088(3)
Ni(2)–O(14)	2.091(3)	Ni(2)–O(12)	2.103(3)
O(5)–Ni(1)–N(3)	170.95(1)	O(5)–Ni(1)–O(1)	90.66 (2)
N(3)–Ni(1)–N(1)	96.38(2)	O(5)–Ni(1)–O(11)	82.19(1)
N(3)–Ni(1)–O(11)	91.80(2)	N(1)–Ni(1)–O(11)	92.48(1)
O(5)–Ni(1)–O(9)	84.23(1)	N(3)–Ni(1)–O(9)	89.05(1)
N(1)–Ni(1)–O(9)	173.90(2)	O(11)–Ni(1)–O(9)	90.13(1)
O(5)–Ni(1)–O(10)	91.71(1)	N(3)–Ni(1)–O(10)	94.38(2)
N(1)–Ni(1)–O(10)	86.54(2)	O(11)–Ni(1)–O(10)	173.81(2)
O(9)–Ni(1)–O(10)	90.27(1)	O(1)–Ni(2)–N(2)	171.96(1)
O(1)–Ni(2)–N(4)	90.89(2)	N(2)–Ni(2)–N(4)	93.15(2)
O(1)–Ni(2)–O(13)	80.99(1)	N(2)–Ni(2)–O(13)	91.77(2)
N(4)–Ni(2)–O(13)	94.20(1)	O(1)–Ni(2)–O(14)	86.31(1)
N(2)–Ni(2)–O(14)	90.17(1)	N(4)–Ni(2)–O(14)	174.84(2)
O(13)–Ni(2)–O(14)	89.64(1)	O(1)–Ni(2)–O(12)	91.48(1)
N(2)–Ni(2)–O(12)	95.59(1)	N(4)–Ni(2)–O(12)	88.14(1)
O(13)–Ni(2)–O(12)	172.14(1)	O(14)–Ni(2)–O(12)	87.60(1)

Table S2. Related parameters of O–H···O hydrogen bonds in **1**. [1]

D–H···A	D–H (Å)	H···A (Å)	D···A (Å)	∠ D–H···A (°)
O(9)–H(9A)···O(4)$_{i}$	0.83	1.87	2.675(1)	163
O(9)–H(9B)···O(7)	0.83	2.07	2.871(1)	163
O(10)–H(10A)···O(6)	0.83	1.94	2.752(1)	164
O(10)–H(10B)···O(2)$_{ii}$	0.83	1.94	2.726(1)	157
O(11)–H(11A)···O(3)$_{i}$	0.83	1.91	2.668(1)	153
O(11)–H(11B)···O(8)	0.82	2.41	3.094(1)	141
O(12)–H(12A)···O(2)	0.83	1.94	2.689(1)	150
O(12)–H(12B)···O(6)$_{iii}$	0.83	1.91	2.713(1)	163
O(13)–H(13A)···O(7)$_{iv}$	0.83	1.85	2.689(1)	178
O(13)–H(13B)···O(4)	0.84	2.38	3.170(1)	157
O(14)–H(14A)···O(8)$_{iv}$	0.83	1.89	2.705(1)	169
O(14)–H(14B)···O(3)	0.83	1.98	2.799(1)	172
O(15)–H(15A)···O(12)$_{v}$	0.91	2.09	2.869(1)	143
O(15)–H(15B)···O(7)$_{vi}$	0.94	1.87	2.719(1)	150
O(16)–H(16)···O(15)	0.84	2.00	2.610(1)	129
O(17)–H(17C)···O(3)$_{ii}$	0.96	2.22	2.862(1)	123
O(18)–H(18)···O(16)	0.84	2.23	2.813(1)	127

[1] Symmetry transformations used to generate equivalent atoms: i = x,–y–1, z+1/2; ii = x–1,–y–1, z+1/2; iii = x,–y, z+1/2; iv = x–1,–y, z+1/2; v = x–1/2, y+3/2, z–1; vi = x–1/2,–y+1/2, z–1/2

Table S3. Bond lengths (Å) and angles (°) around Zn(II) ion in **2**.[1]

Zn(1)–O(1)	1.997(9)	Zn(1)–N(1)	2.024(1)
Zn(1)–N(2)	2.025(1)	Zn(1)–O(3)i	2.030(9)
O(1)–Zn(1)–N(1)	110.05(4)	O(1)–Zn(1)–N(2)	108.05(4)
N(1)–Zn(1)–N(2)	110.25(4)	O(1)–Zn(1)–O(3)i	102.29(4)
N(1)–Zn(1)–O(3)i	117.72(4)	N(2)–Zn(1)–O(3)i	107.90(4)

[1] Symmetry transformations used to generate equivalent atoms: i x,-y+1/2,z-1/2.

Table S4. Related parameters of O–H···O hydrogen bonds in **2**. [1]

D–H···A	D–H (Å)	H···A (Å)	D···A (Å)	∠ D–H···A (°)
O(5)–H(5A)···O(8)	0.83	1.95	2.766(1)	167
O(5)–H(5B)···O(1)i	0.79	2.08	2.867(1)	171
O(6)–H(6A)···O(7)	0.81	1.93	2.725(1)	171
O(6)–H(6B)···O(4)ii	0.83	2.07	2.854(1)	159
O(7)–H(7A)···O(9)ii	0.88	1.95	2.818(1)	170
O(7)–H(7B)···O(5)iii	0.82	1.99	2.804(1)	174
O(8)–H(8A)···O(9)	0.84	1.90	2.735(1)	178
O(8)–H(8B)···O(3)iv	0.82	2.10	2.884(1)	160
O(9)–H(9A)···O(2)	0.81	1.96	2.771(1)	175
O(9)–H(9B)···O(6)	0.81	1.95	2.757(1)	176

[1] Symmetry transformations used to generate equivalent atoms: i = –x–1,–y–1,–z; ii = –x, y+1/2,–z+3/2; iii = –x,–y,–z; iv = –x–1, y+1/2,–z+3/2

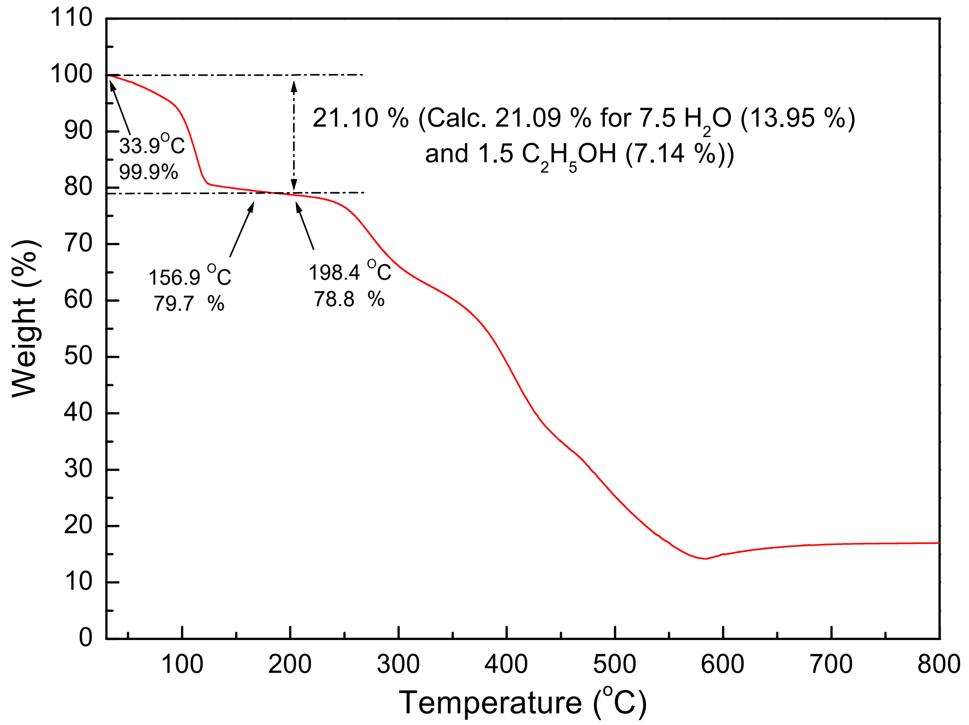

(a)

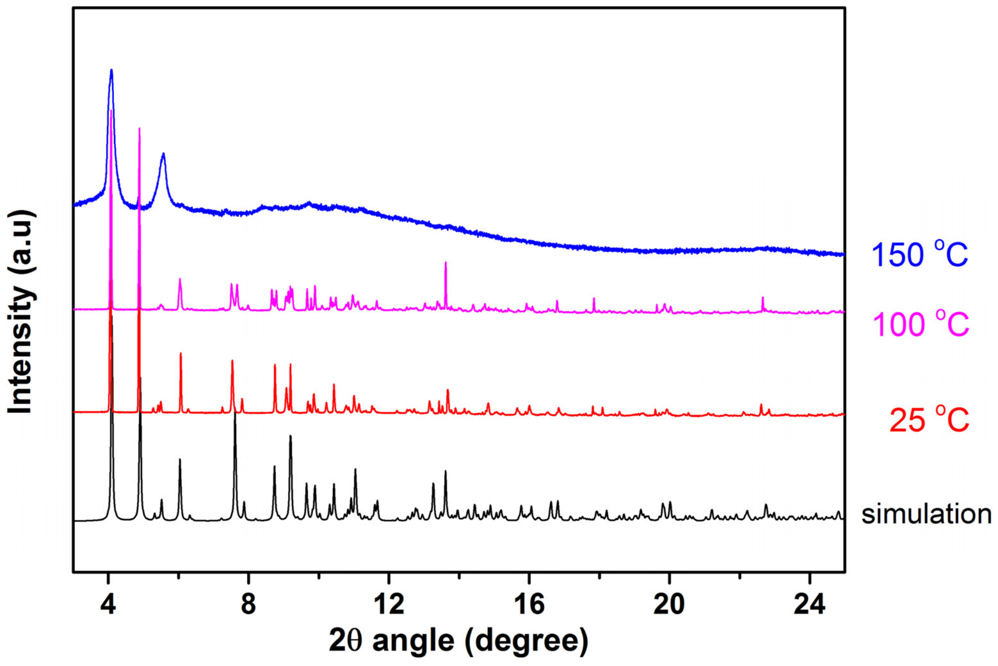

(b)

Figure S1 (a) Thermogravimetric (TG) measurement of **1**. (b) Powder X-ray diffraction patterns at RT and selected temperatures and its simulation from single-crystal diffraction data of **1**.

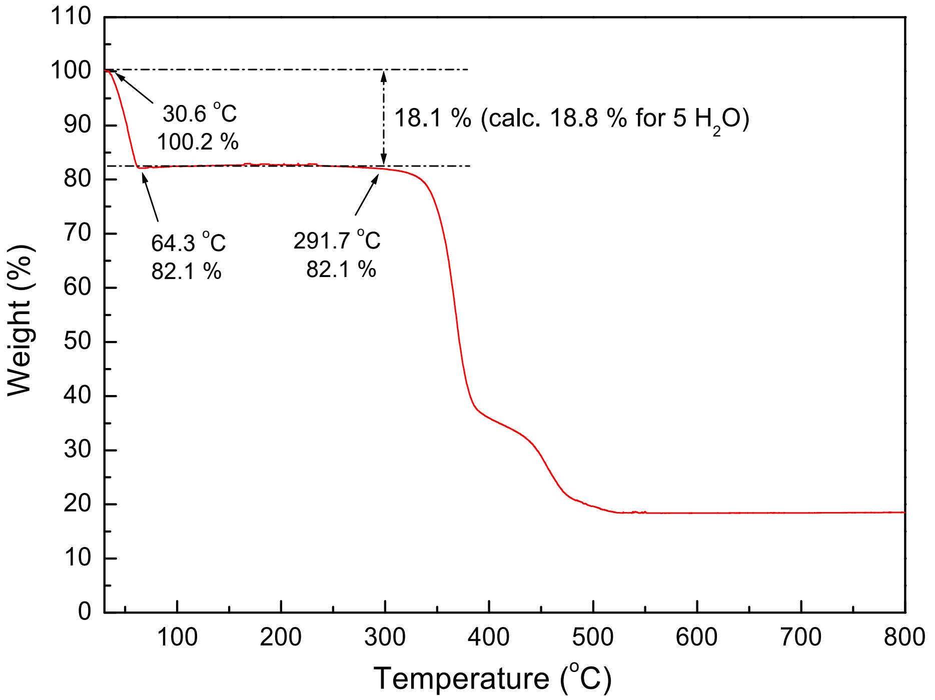

(a)

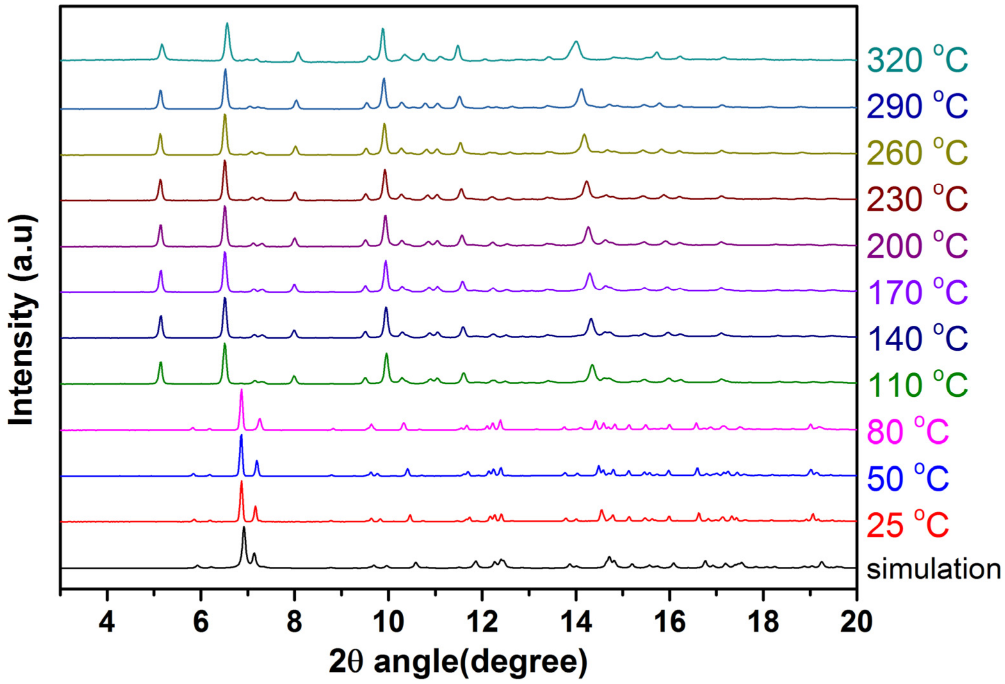

(b)

Figure S2 (a) Thermogravimetric (TG) measurement of **2**. (b) Powder X-ray diffraction patterns at RT and selected temperatures and its simulation from single-crystal diffraction data of **2**.
